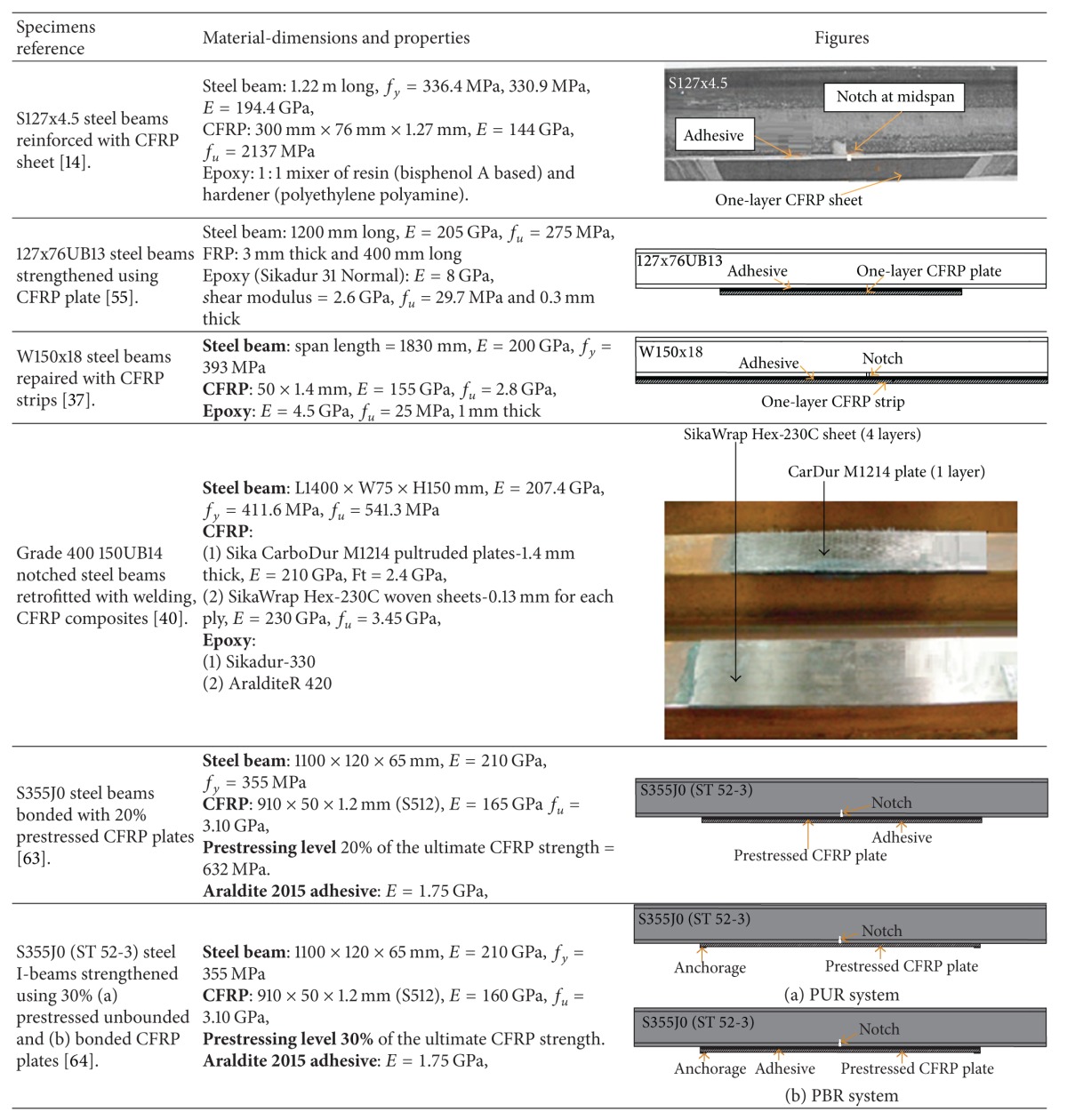# A Review on Strengthening Steel Beams Using FRP under Fatigue

**DOI:** 10.1155/2014/702537

**Published:** 2014-08-27

**Authors:** Mohamed Kamruzzaman, Mohd Zamin Jumaat, N. H. Ramli Sulong, A. B. M. Saiful Islam

**Affiliations:** Department of Civil Engineering, University of Malaya, 50603 Kuala Lumpur, Malaysia

## Abstract

In recent decades, the application of fibre-reinforced polymer (FRP) composites for strengthening structural elements has become an efficient option to meet the increased cyclic loads or repair due to corrosion or fatigue cracking. Hence, the objective of this study is to explore the existing FRP reinforcing techniques to care for fatigue damaged structural steel elements. This study covers the surface treatment techniques, adhesive curing, and support conditions under cyclic loading including fatigue performance, crack propagation, and failure modes with finite element (FE) simulation of the steel bridge girders and structural elements. FRP strengthening composites delay initial cracking, reduce the crack growth rate, extend the fatigue life, and decrease the stiffness decay with residual deflection. Prestressed carbon fibre-reinforced polymer (CFRP) is the best strengthening option. End anchorage prevents debonding of the CRRP strips at the beam ends by reducing the local interfacial shear and peel stresses. Hybrid-joint, nanoadhesive, and carbon-flex can also be attractive for strengthening systems.

## 1. Introduction

The collapse of structural elements due to fatigue is extremely expensive and may be catastrophic in terms of human life and damage. The fatigue process can be defined as the accumulation of damage at a localized region as a result of cyclic loads, which leads to the formation of a crack that eventually propagates through the material. When a crack grows to a size for which the net-section is inadequate to carry the imposed load, rapid fracture takes place. Fatigue failure of steel girders is one of the most significant problems affecting the remaining service life of existing steel structures like bridges. The application of fibre-reinforced polymer (FRP) composites to increase the fatigue strength of damaged steel girders is a promising technique that offers an attractive substitute to traditional approaches like steel plating.

In recent decades, the strengthening of the steel components of structures has become essential for structural retrofits. FRP strengthening seems to be an effective alternative for such retrofitting. A survey of European railway administrations covering nearly 220,000 bridges across Europe [1] has shown that almost 22% of the bridges are constructed with metal. Among them, 28% of the metallic bridges are over 100 years old, and 40% are between 50 and 100 years old. The federal highway administration of the US Department of Transportation, 2005 [2], estimated that among the almost 200,000 metallic highway bridges in the US, around 40% are either “structurally deficient” or “functionally obsolete.” Deterioration is mostly due to accidental vehicular impacts, cyclic loads. Bridge structures that support moving trains are subjected to high stresses caused by extreme vibrations and dynamic deflections that are far greater than ever before [2]. Moreover, the fatigue life of steel structures could be extensively reduced due to earthquake loadings [3]. The dynamic reaction of railway bridges is also influenced by various factors including the train speed, natural frequency of the bridge, and the bridge and carriage lengths [4]. The I-35W Bridge over the Mississippi river collapsed on 1 August 2007, resulting in 13 dead and 145 injuries, and damaged 111 vehicles. The National Transportation Safety Board [5] cited on the collapse of the I-35W Bridge that a design fault, improper maintenance, and additional weight, because of increasing traffic volume and heavy traffic, caused the fatigue failure. The fatigue damage reported in Japan has been caused mainly by cyclic secondary stress or distortion-induced stress [6].

Fibre-reinforced polymer possesses outstanding advantages as a structural material, including high strength, anticorrosion properties, and high durability and is able to restore the lost capacity of damaged structures [7, 8]. FRP sheets/strips are also effective in the strengthening of steel structural elements to extend their fatigue lifetime and reduce crack propagation [9–11] if galvanic corrosion is prevented and sufficient bond is provided [12, 13]. Recent strengthening projects in the United States, Switzerland, the United Kingdom, and Japan showed that there was a great potential for using carbon fibre-reinforced polymer (CFRP) to retrofit steel structural elements [14–16]. CFRP materials have been used for marine structures [17, 18] and aerospace parts [19] and have expanded to wood [20], masonry [21], steel/concrete composite structures [22], and so on. It is more frequently used for rehabilitation and strengthening of steel structures than other FRP materials due to its high strength. In any case, CFRP is very tolerant to fatigue damage [23, 24]. Basalt-fibre-reinforced polymers (BFRP) have been increasingly considered in civil infrastructures, because of low cost and their excellent chemical and mechanical properties [25, 26].

The Acton bridge in England [27] and the Ashland bridge and 7838.5S092 bridge in the USA were retrofitted by applying CFRP elements to the bottom of the girders; the stress reduction on the original materials was observed to increase the fatigue life [28]. Several case studies of existing steel railway bridges are presented in [29, 30]. Monitoring data was used for dynamic response analysis and fatigue life evaluation. It was observed that the stringers and the cross beams run a high risk of fatigue damage. The weakest point in the reinforcing system of FRP elements to steel joints is the bond of the adhesive [31–33]. The successful implementation of FRP composites of the strengthening systems is dependent upon the quality and integrity of the steel-composite joint and the effectiveness of the epoxy adhesive used [34]. The fatigue performance of the CFRP reinforcement details is necessary to check if, under realistic severe spectrum loading, the adhesive joint performs better than the common fatigue sensitive details found on steel bridge girders, in accordance with Eurocode 3 categories 36* and 50* [35] or American Association of State Highway and Transportation Officials (AASHTO) categories E′ and E [36].

The application of FRP composite for strengthening steel bridges and structural elements has become an efficient option to meet the increased cyclic loads or repair due to corrosion or fatigue cracking. Therefore, the objective of this review is to explore the potential FRP reinforcing techniques to care for fatigue damaged steel structural elements. This paper reviews the research related to CFRP/steel strengthening techniques under fatigue. Detailed knowledge and explanation of the existing research concerning the fatigue behaviour of HM, HS, UHM, and prestressed CFRP and SW-BFRP strengthened steel structures are provided. The study also covers the surface treatment techniques, adhesive curing, and support condition under cyclic loading including fatigue performance, crack propagation, and failure modes with FE simulation of the steel bridge girders and structural elements. Future research gaps and recommendations are indicated accordingly.

## 2. Surface Preparation and Treatment for Steel Beam Strengthening

### 2.1. Types of Notch

**Figure 1:**
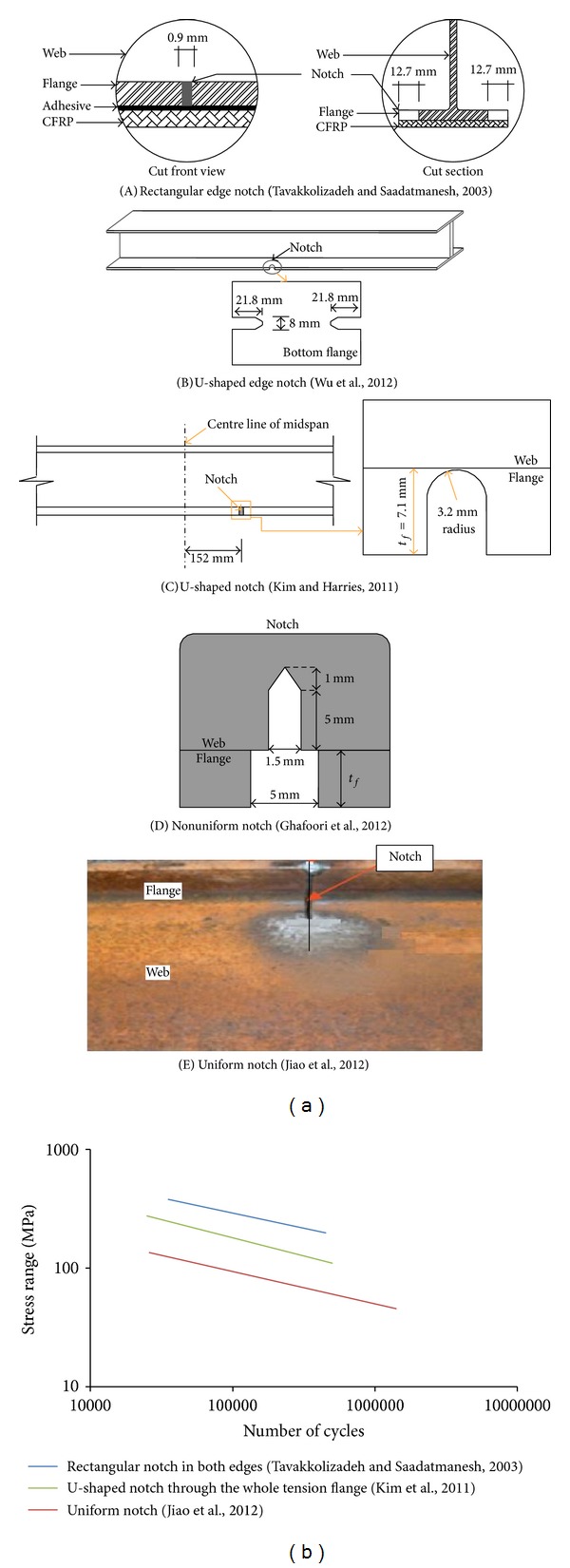
(a) Types of notch in steel beam. (b) Stress behaviour in fatigue for different notch categories [14, 37, 40].

To simulate the actual damage caused by corrosion and the expansion of fatigue cracks, several researchers intentionally created notches of different geometry in midspan or other positions on the tension flange of the beams (Figure 1(a)). In addition, the notch assists like a stress concentrator [37] in the damage-sensitive regions [38, 39] to commence a vertical crack at the steel web. The different types of notch can be categorized as follows:

- rectangular notch on both edges (Figure 1(A)),
- U-shaped notch on both edges (Figure 1(B)),
- U-shaped notch through the whole tension flange (Figure 1(C)),
- nonuniform notch (Figure 1(D)),
- uniform notch (Figure 1(E)).

Usually, the researchers cut a notch at midspan in the tension flange of the steel beams except Kim and Harries [37]. To initiate the debonding of CFRP, which was aimed at propagating towards the right support, they created a notch through the entire tension flange at a position 152 mm to the right of midspan of the beams. Jiao et al. [40] welded the cut along the tension flange soffit using the shielded metal arc welding (SMAW) approach.

The notch (C) through the whole tension flange is sensitive to fatigue compared to the side notches (A) and (B). Accordingly, notches (D) and (E), which go through the whole tension flange with part of the web in midspan, are more sensitive to fatigue damage as the damage occurs suddenly.

From the above categories of creating the notch, it has been revealed that when the notch spreads through the flange as well as the web of the steel beam, the propagation of the cracks exists in the notch line of the flange and the web. Brittle fracture can happen in the case of fatigue. This is injurious for the structural element as no warning is given before failure. In addition, notches only created in the flange can expect a retarding fracture with prior warning. However, if the notch is given in the whole tension flange, there is also the possibility of brittle fracture under heavy repeated load. Therefore, for observing the proper fatigue damage, a rectangular and U-shaped notch on both edges at midspan may be competently incorporated for the development of a standardized test.  Figure 1(b) illustrates the stress characteristics of a strengthened steel beam under cyclic loading for incorporation of different notches. The S-N curves show that the uniform notch comprising whole flange and web has the least stress compared to others. This clearly demonstrates more fatigue life when the side rectangular notch is incorporated in the flange.

### 2.2. Welding Cover Plate and Stiffener

**Figure 2:**
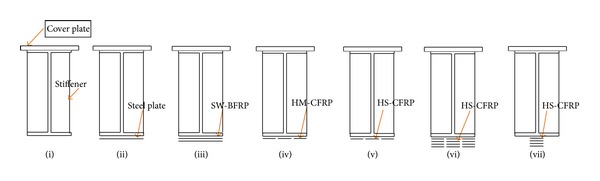
Strengthening technique with plate configuration [38].

To provide the equivalent of a concrete slab that typically exists in bridges for preventing compression flange buckling, Wu et al. [38] attached a steel cover plate with welding on the outside surface of the top flange of the steel beams (Figure 2). They also welded web stiffeners on both sides of the web at the loading and supporting points. Web stiffeners assist in preventing web crippling at the midspan section [41]. In order to provide lateral stability of the steel beams, Kim and Brunell [42] used stiffeners that were welded at the supporting points. The adhesively bonded steel stiffeners to the flanges and webs on both sides of the beam extensively retarded local buckling of the steel beams [43]. Siddique and El Damatty [44] showed that the use of glass fibre-reinforced polymer (GFRP) composites enhances local buckling behaviour of wide flange steel beams, which is effective, especially for the case of slender beams. The addition of GFRP plates to the compression flange of a steel beam increases both the load carrying capacity and the deflection at failure. The improvement in the load capacity is independent of the web dimensions of the beams for both plastic and slender beams. The study ignored the use of a stiffener. The mode of failure of the retrofitted slender beams ranges from elastic buckling of the system to GFRP rupture when the thickness of the GFRP is varied from 6.35 mm to 19.0 mm. As the GFRP thickness is significant in their study it could well improve the local bucking.

### 2.3. Prevention of Galvanic Corrosion

Although CFRP is a noncorrosive substance, when carbon fibres are in contact with steel, they can form a galvanic cell. To increase the fatigue strength of bridge girders and long-term durability of CFRP reinforcement in a steel structure, the prevention of galvanic corrosion is necessary. Furthermore, to rule out the galvanic corrosion, the flow of corrosion needs to be prevented. This may be accomplished by insulating the different metals from one another or through preventing a continuous link of electrolytic solution between the two by coating with a water resistant sealant [45]. It is obvious that if the two different metals are not in contact, galvanic corrosion cannot occur [46]. Tavakkolizadeh and Saadatmanesh [47] examined the galvanic corrosion between carbon and steel for various thicknesses of adhesive coating in different electrolytes, such as seawater and deicing salt solution. The thin coating effect of adhesive (0.25 mm) was found to be substantial as was the sizing applied to the fibres. Furthermore, a thicker adhesive between the surfaces of the CFRP and steel was observed to suggestively slow the rate of corrosion of the steel.

Mitigating galvanic corrosion of the CFRP-steel composite can be achieved by the selection of an adhesive with good quality isolation properties [15] or by using a thicker epoxy, water resistant sealant, or nonconductive layer plus a sealant, or by bonding a GFRP sheet before applying the CFRP layer onto the steel surface [17, 48–50]. Hollaway and Cadei [51] installed a polyester drape veil to provide insulation between the steel and the carbon fibre for preventing direct contact between them. Fibreglass or an epoxy film was considered to provide effective insulation. In addition, a monitoring programme was initiated to identify the cathodic sites so that galvanic corrosion damage could be mitigated or stopped [34].

### 2.4. Surface Treatment

The reliability of the joint is highly dependent upon the surface treatment processes for bonding the fibre-reinforced composite to the steel structural elements [52]. The surface preparation and the strength of the applied CFRP overlay can significantly affect the fatigue performance [53]. For assessing the effect of the CFRP strengthening technique on the fatigue strength, Jiao et al. [40] used a grinder to remove the corrosion as well as to level the weld area on each steel beam soffit before applying the adhesive. To obtain a clean, rough, and chemically active surface, Wu et al. [38] treated the surface of the tension flange using a grinding wheel to reinforce with CFRP for the fatigue test. The surfaces of the tension flange and composite plates were then washed with acetone.

Tavakkolizadeh and Saadatmanesh [14] used a sand blaster meticulously by number 50 glass bids and washed with saline solution just prior to the application of the composite sheet to prevent oxidation. The study by Teng et al. [54] showed that sand blasting was the most effective surface treatment. Prior to bonding the CFRP strips to the beams, Kim and Harries [37] used a 1500 sfpm (surface feet per minute) belt sander and a 40 grit zirconia alumina belt. This ensured a sound, slightly striated surface to bond the CFRP strips. The adhesive layer thickness was approximately 1 mm.

Deng and Lee [55, 56] found that the tips of the FRP plates must be finished smoothly using sandpaper before the attachment of the plate to the steel beams. However Schnerch et al. [57] disagreed with Choudhury [58], as they contended that preparing the surface with a hand grinder followed by sandpapering reduces the bonding ability of the surface. However, a chemically active steel surface that is free from contaminants is essential to enhance the chemical bond between the adhesive and the metallic surface. Brushing, ultrasonic, or vapour degreasing systems are claimed to be the most efficient to remove oil and other potential surface contamination, especially when adequate solvents are used [59]. Contamination may then be removed using the excess solvent, rather than simply redepositing it on the steel surface as the solvent evaporates.

The most efficient means of achieving a high-energy surface of the steel is by grit blasting [51]. Grit blasting with angular grit removes the inactive oxide and hydroxide deposits by cutting and deformation of the base material. The grit size also affects the surface profile of the steel. Harris and Beevers [60] stated that finer particles created a smoother surface than coarser grit particles and smoother surfaces exhibited higher adhesive-steel surface bonding. In addition, the surface profile of the steel was not influenced on the long-term durability [34]. After grit blasting, solvents may be used to wash and clean the steel surface without resulting in poor bonding [61, 62].

## 3. Fatigue Strengthening Techniques for Steel Beams

The modulus of elasticity, tensile strength, shape, and configuration of FRP composites of an adhesively bonded joint play an important role in respect of the fatigue strength and lifetime of reinforced steel beams and bridge girders. A number of researchers have investigated reinforced steel beams with different FRP strengthening techniques and compared their fatigue performance. A summary of the reinforcing technique of steel structures using fibre-reinforced composites is provided in Table 1.

Furthermore, Wu et al. [38] investigated eight artificially damaged H350 × 175 steel beams, including one unstrengthened and seven strengthened with welded steel, SW-BFRP, high modulus CFRP (HM-CFRP), and high strength CFRP (HS-CFRP) plates using Sikadur-30 Normal epoxy adhesive. The plate configuration of the strengthening technique used by Wu et al. [38] is shown in Figure 2. The elastic modulus, ultimate strain, tensile strength, and shear strength of the epoxy were 2.627 GPa, 1.5%, 31.7 MPa, and 14.4 MPa, respectively. An anchorage system was applied at below the point load and at the end of the fibre-reinforced composite plates. The HM-CFRP has the most excellent strengthening effects and SW-BFRP is the best strengthening material on the basis of the cost-performance ratio [38].

Basalt-FRP (BFRP) composites show synthetical advantages in structural strengthening, seismic rehabilitation, and serving as new structural materials [26, 65]. However, the relatively low modulus of BFRP may not satisfy the stiffness requirement of some structures. Therefore, to obtain higher performance, steel-wire- (SW-) BFRP can be made from hybridization of BFRP with steel wires (SW) or CFRPs [26, 66].

A 210 ft × 26 ft three continuous span rolled steel bridge girder in Guthrie County, Iowa, on state highway 141 was strengthened using externally posttensioning CFRP rods [67]. The anchorage systems were bolted to the webs of the steel girders. The proposed prestressed unbounded reinforcement (PUR) system [64] can be applied as an alternative to adhesively bonded FRP reinforcement, mainly when there is concern about the effects of water, moisture, high ambient temperatures, and high cycle loading on the glue between the FRP and the steel. Vatandoost used 14%, 15%, 35%, 0%, and 37% prestressed CFRP plates to investigate the fatigue behaviour of five W310 × 74 steel beams Vatandoost [68], in which the 14%, 15%, and 35% prestressed CFRP plates were bonded to the inner side of the bottom tension flange, and the 0% and 37% prestressed plates were attached to the cover plate. A prestress FRP composite patch is strongly suggested to maximize the effectiveness of the adhesively bonded patch on the steel element [69] and fatigue strengthening [70]. The FRP prestressing system developed at Empa was used for the CFRP strips for applying a direct tensile force against an external reaction steel frame by jacking. Vatandoost and others [68, 71–73] discussed more details concerning the prestressing procedure.

Recently, the carbon-flex, that is, carbon fibre hybrid-polymeric matrix composite (CHMC) strengthening technique was developed by Zhou and Attard [74], which is a carbon fibre-based composite manufactured using the latest hybrid-matrix technique involving amino-based polymeric composites to provide necessary damping and high strength sustainability of the carbon fibrous element. Recently, Zhou and others [74–76] indicated the enormous potential of carbon-flex as a strengthening substance to subsequently prevent higher damage or catastrophic failure of structures.

The majority of fatigue problems arise from poor detailing or careless fabrication, rather than inaccurate materials selection [77]. Schnerch and others [15, 52] reported that the bonding mechanisms of FRP strengthened steel structures are different than concrete structures. In addition, high bonding stresses occur in steel structures to meet the strengthening requirements [34]. Any violation of fabrication tolerances can unpredictably change the fatigue behaviour and lead to a very scattered fatigue life [73].

## 4. Adhesive Curing

If a bridge or long span structure is retrofitted with CFRP strips, it is generally not economic to close it to traffic during the adhesive curing time, which can take up to 48 hours. During this time, the epoxy adhesive is subject to repeated loading from the traffic. The Concrete Society [78] recommended that the change in the epoxy properties caused by the repeated load during the curing time is expected to be small, perhaps a 10% decrease in the strength of the fully cured structural elements. Nikouka et al. [79] studied the improvement in the strength and stiffness of strengthened steel beams with CFRP subjected to repeated loading during the early age curing of the epoxy adhesive. Five pairs of 127 × 76UB13 type steel beams, each 1.2 m long, were strengthened with a 0.98 m long single K13710 ultra-high modulus CFRP plate, attached to the bottom tension flange. A cyclic load was applied to the five specimens with 0.25 Hz frequency and was continued for up to around 48 hours. The study reported that during the curing of the epoxy, the cyclic loading would affect the final stiffness and failure load of the strengthened beam when the highest cyclic load was larger than 42 kN. Moreover, the bond would fail to develop if the shear deformation in the epoxy layer during the cure is too large. They also recommended that it was prudent to limit the shear stress in the epoxy to a maximum of 1 MPa.

Bourban et al. [80] indicated a clear advantage from the epoxy adhesive curing at high temperatures (about 93°C) during the initial cure (10–20 minutes). The resulting bond is stronger, tougher, and more durable when subject to unfavourable environments [81]. With the intention of retrofitting steel bridges open to traffic during the adhesive curing period, Moy [27] investigated the effect of repeated loading on the curing of the epoxy. The results confirmed a progressive stiffness increase of the reinforced component as the epoxy cured. Furthermore, the beams subject to higher loads during curing did not develop the full bond of the epoxy adhesive. The tests performed showed that cyclic loading at higher load levels reduced the ultimate capacity of the strengthened beams [41]. In addition, the vibration of the traffic during the curing of the adhesive causes a progressive reduction in the fatigue lifetime with increasing strain level [82, 83].

Zhang et al. [84] proposed an innovative method involving preimpregnation (prepreg) advanced composites and a compatible epoxy film for retrofitting steel railway bridges open to traffic during the curing period of the epoxy adhesive. The strengthening system was made from unidirectional HM- and UHM-CFRP preimpregnations that were cured on site under vacuum assisted pressure. Two cure temperatures were used: 65°C for 16 hours and 80°C for 4 hours. A GFRP prepreg layer was placed between the CFRP patch and the steel element. The beams were initially induced by vibration forces and then loaded to failure. From the experimental results it was observed that despite slight damage at the adhesive level, the proposed technique prevented severe brittle failure of the composite beam.

## 5. Support Condition and Instrumentation

**Figure 3:**
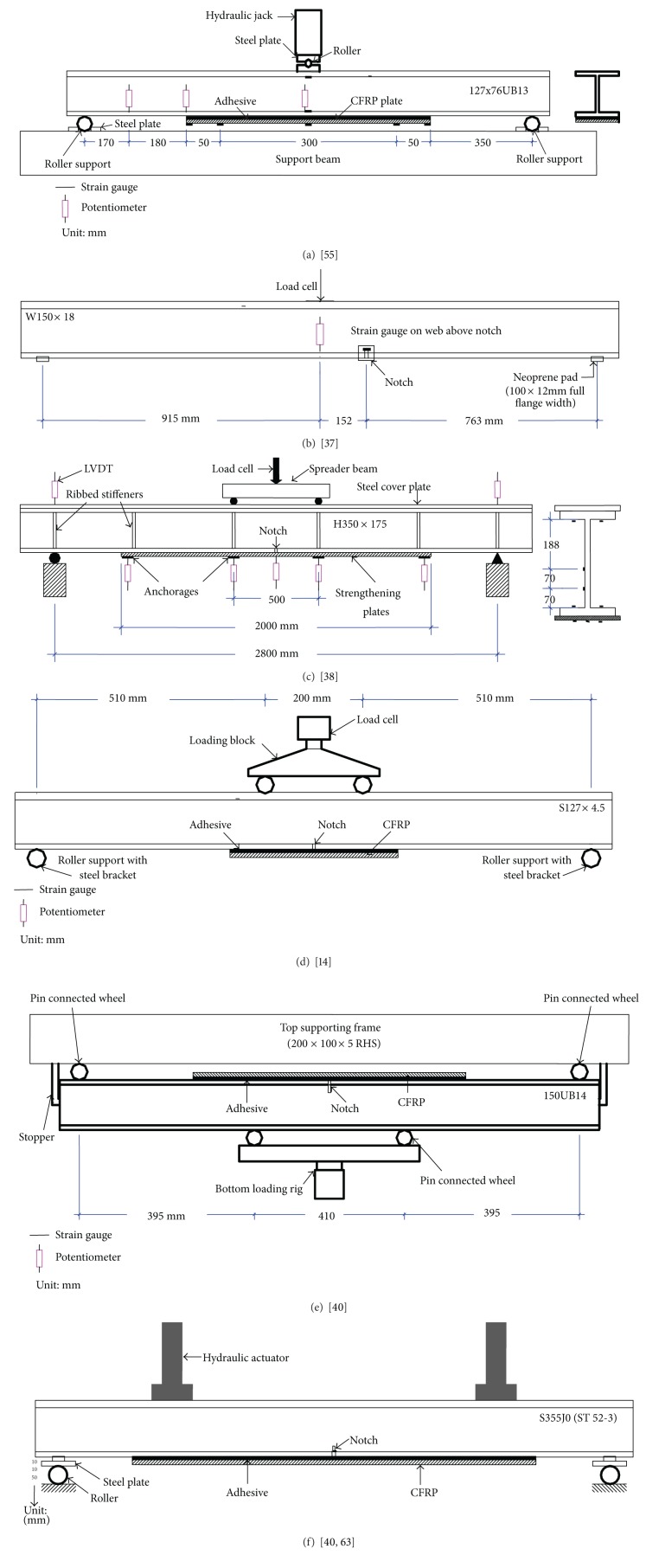
Support condition with fatigue test setup.

Different support conditions have been adopted by different researchers for the fatigue test programmes of steel beams, as shown in Figure 3. Deng and Lee [55] tested the fatigue strength of nine reinforced steel girders by a servo-hydraulic Dennison testing machine, using a three-point bending setup as a simply supported beam (Figure 3(a)). The specimens were supported on two rollers but were restrained from any sideways movement. The loading block had two steel plates, each with a counter seat and a roller in between. Deflections were measured at three locations by means of potentiometers. Five 2 mm and two 5 mm long strain gauges were used to investigate the crack initiation as well as the effect of crack growth on the stress field in the girder. All the data were recorded using a data logger. Studies on the fatigue of double sided reinforcement subjected to tension and full-scale bridge girders retrofitted with CFRP plates under three-point bending were conducted at the University of Delaware [85]. Kim and Harries [37] used a neoprene rubber pad between the support and beam to reduce the concentration of stress, as shown in Figure 3(b). In all cases, the CFRP plates remained fully bonded to the steel element. The results suggested excellent fatigue behaviour of the reinforced elements.

Wu et al. [38] tested the strengthened H350 × 175 steel beams under constant amplitude cyclic load using 4 Hz frequency as a simply supported mode and four-point bending, as shown in Figure 3(c). The load was measured by the loading cell of a MTS system. To prevent any movement of the specimen during the test, Tavakkolizadeh and Saadatmanesh [14] used tie down brackets to the roller supports. The loading blocks were designed using a counter seat for the compression flange in order to prevent their movement during the experiments. The loading setup is shown in Figure 3(d). The specimens were tested using various constant stresses ranging between 69 and 379 MPa (*R* = 0.1) and a frequency of 5 and 10 Hz. Vertical displacements can be measured by linear variable displacement transducers (LVDT) with a range of ±50 mm [38] and ±75 mm [14].

Jiao et al. [40] conducted fatigue tests under load control with 7 Hz on strengthened steel beams with a 4-point bending rig using a MTS-810 testing machine, which contained the top supporting frame and bottom loading beam as shown in Figure 3(e). Two 12 mm thick steel plates were welded to the midspan of the top supporting frame and the bottom loading beam. Four pin connected wheels were employed at the supporting and loading points that could freely rotate during the fatigue tests. Two screw-fixed stoppers were used on both sides of the bending rig to prevent the test specimen from changing position during the experiment. The bottom loading rig was designed using a three-pin system to ensure that the load was distributed between the two loading points. Using SHOWA strip strain gauges, the ultimate load, displacement, number of cycles, and corresponding strains of each cycle were recorded using the National Instrument NI 9237 Compact Data Acquisition system.

To fatigue test the reinforced metallic beams using prestressed FRP, Ghafoori et al. [64] used a pulsator P960 oil hydraulic test machine with a four-point bending setup. The lubricated rollers of 5 cm diameter at the supports and a steel plate were employed between the beam and rollers to distribute the load properly (Figure 3(f)). Ghafoori et al. [63] used a 3D ICS (image correlation system) to measure the crack length and the corresponding strain deformation at the crack tip area. The measurement window of the ICS was set at 65 mm × 65 mm. The calibration details and the use of the ICS can be found in [87–89]. The field signature method (FSM) is also effective for detecting and monitoring cracks on steel structures [90].

## 6. Finite Element (FE) Simulation

The finite element method (FEM) is an acceptable approach for analysing structures using software. In practice, the FE simulation is developed to validate the fatigue strength of the experimental or analytical results.

**Figure 4:**
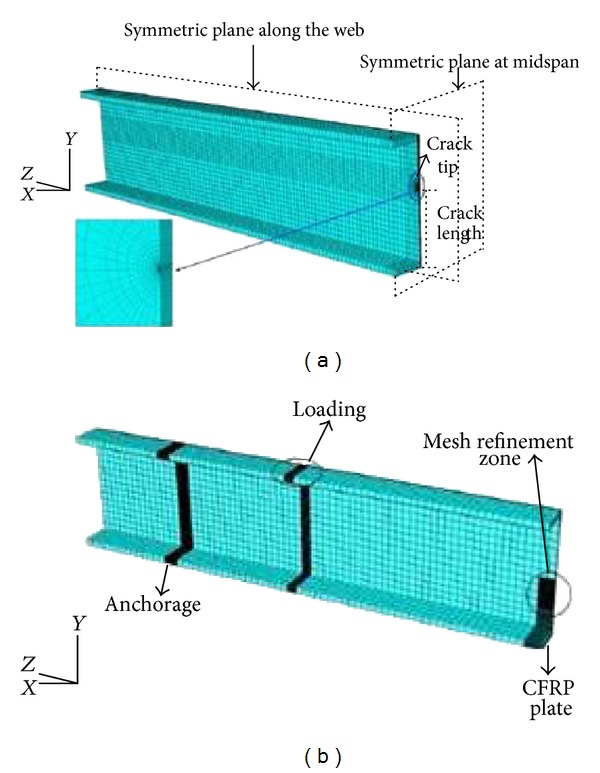
(a) A geometrical model using ABAQUS in the FE analysis and (b) the mesh refinement around the loading, anchorage, and crack zones [64].

Based on the surface crack widening energy release rate [91] using an elementary material strength theory [92] and G*-integral [93], an analytical approach was introduced by Ghafoori and Motavalli [87] to estimate the stress intensity factors (SIF) of a cracked steel I-beam. The fatigue rehabilitation of steel structures is usually expected to decrease the value of SIF at the tip of the crack and, as a result, enhance the postcrack fatigue life [94]. Ghafoori et al. [64] proposed an analytical method using the experimental test data (the external bending moment, the length of the crack, and the corresponding strain imposed on the CFRP strip under the cracked segment) and produced the SIF. They used ABAQUS software (version 6.8) to analyse the FE model of the steel beams to validate the results. The geometrical model and more mesh refinement around the loading, anchorage, and cracked sections are shown in Figure 4. The method was developed to assess the sufficient level of the CFRP prestressing to arrest the fatigue crack growth (FCG). Moreover, the method was used to study different active, semiactive, and passive crack modes with a loaded reinforced beam. Several factors have been considered including crack propagation, excitation frequency, and structural damping on the life of the FCG [95].

Using the concept of fracture, the fatigue crack propagation (FCP) model was proposed by Xiulin and Hirt [96]. This was extended to the FCP of a cracked metallic element retrofitted with adhesively bonded composite patches in the study of Wang and Nussbaumer [97].

According to the Paris-Erdogan crack growth law [98], a linear elastic fracture mechanics (LEFM) model was employed to predict the effects of peening treatments on the fatigue performance of welded steel structures [99] and to confirm the effectiveness of the prestressed CFRP strips [68]. Ghafoori et al. [63] introduced a methodology for a damaged beam with a specific crack length that is subjected to a certain cyclic load based on the fracture mechanics (FM) theory to estimate the adequate prestressing level by which the crack propagation is detained. Some strengthened beams were tested under various cyclic loading ranges and the experimental results showed excellent agreement with the developed fracture model.

In fracture mechanics, based on progressive damage modelling methods to predict the fatigue life, the rate of crack growth is related to the SIF [98] or strain energy release rate [100–103]. In the case of adhesively bonded joints, a damage shift parameter was proposed to account for the effects of accelerative interaction [102, 103]. Important interaction effects were considered where crack growth acceleration was linked with mean load changes. However, continuum damage mechanics (CDM) models were developed by Lemaitre and Desmorat [104] and modified for the damage formation of microcracks by [105, 106]. CDM models have been used in a damage evolution law for modelling both precracking damage evolution and crack growth for constant and variable amplitude fatigue [107]. In the case of bonded joints, Wahab et al. [108] compared both the FM and the DM methods to predict the fatigue strength of adhesively bonded CFRP double lap joints. They verified that the developed CDM approach compared favourably with a FM method for constant amplitude fatigue (CAF). The FM and DM based fatigue life prediction of bonded single lap joints (SLJs) subjected to different types of variable amplitude fatigue (VAF) loading was analysed by Shenoy et al. [109].

**Figure 5:**
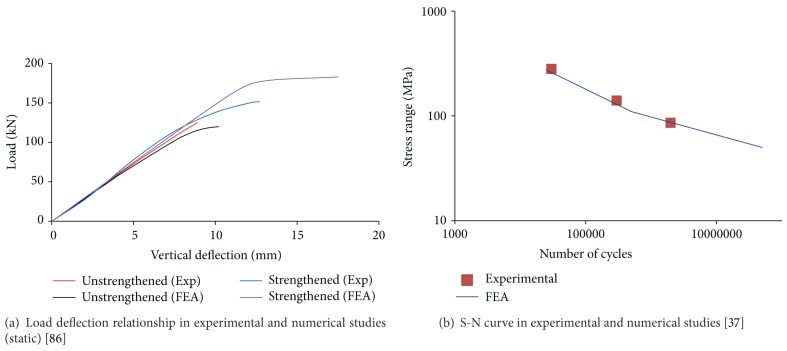
Comparison of experimental and simulation outputs of steel beam.

Kim and Harries [37] developed a three-dimensional (3D) nonlinear finite element model for predicting the fatigue strength of notched steel beams using ANSYS software. The steel section was modelled using 3D structural solid elements (SOLID45); and a linear stress-strain relationship was developed for the CFRP. A nonlinear interface element (COMBIN39) with two nodes was applied for modelling the behaviour of the steel-CFRP interface. For the element whose initial relative distance is zero, a bilinear bond-slip relationship was created for them. The study used the strain life method and the concept of Henry's damage theory [110] for the fatigue life prediction of steel beams. The strain life approach is mainly relevant to a member representing significant plasticity induced by hysteretic loads. The theoretical background of this approach is discussed by Bannantine et al. [111]. The deflection behaviour of unstrengthened and strengthened beams is shown in Figure 5(a). Furthermore, a typical S-N curve of strengthened steel beam obtained is shown in Figure 5(b), which was compared with category E in the AISC in the study by [112]. The notch provided for the stress concentrating effect is essentially equivalent to a Category E detail. Apart from this, Youssef [113] developed a model for predicting the linear and nonlinear behaviour including the deflection at midspan, strains of the steel and FRP, failure mechanism, and failure load of rehabilitated steel beams. The model was founded on the solution of the differential equations governing the behaviour of a strengthened steel beam, which includes representation of the shear and peel behaviour of the epoxy adhesive. To validate the predictions of the model, a W-shaped steel beam strengthened using GFRP sheets was experimentally tested and excellent agreement was found between these results.

Zhou et al. [114] adopted the micromechanics based fracture model and the cyclic void growth model (CVGM) for estimating extremely low cycle fatigue (ELCF) fracture of the column-to-beam connections during earthquakes. The model was verified by the experimental results of nine full-scale connection tests. In addition, the refined finite element model was used to simulate the cyclic behaviour of the connection tests, and the CVGM fracture index was calculated using the stress and strain time histories. The number of cycles and the cumulative deformations to ELCF fracture predicted by CVGM agreed well with the experimental results. The existing methodology also showed reasonable good accuracy for predicting the ELCF fracture of column-to-beam connections under inelastic cyclic loadings.

Pipinato et al. [115] used a LEFM method in a probabilistic [116] context to assess the fatigue reliability of steel bridge girders in the presence of seismic loading. This method could enable a better understanding of progressive damage phenomena due to fatigue problems and could give some new insights to increase the remaining fatigue strength of a large number of steel bridges in seismic regions. Colombi [117] developed a suitable plasticity based [118, 119] crack retarding model as an extension of the well-known Newman's model [120, 121] to estimate the reduction of crack opening displacement along with the magnification of the crack growth retardation of the reinforced notched steel plates.

From the abovementioned literature, it is revealed that the simulation in the finite element method can be a vital tool to assist in strengthening beam analysis under fatigue. This is because it eventually decreases the experimental cost in finance and time. Good validation of the simulation with practical experiments ensures the advantages of the strengthening techniques. However, the characteristics of strengthened steel beams under fatigue without using notches are still an interesting area to be explored. This interest can be addressed by FE simulation in a consistent manner.

## 7. Fatigue Performance of Reinforced Steel Beams

A steel structure subjected to repeated load may eventually experience significant fatigue damage during its life. A number of researchers concentrated on fatigue strength and fatigue lifetime prediction of reinforced steel beams and bridge girders. This indicates that there is a need to enhance the fatigue strength and prolong the fatigue lifetime of steel structures with adhesively bonded metal/FRP strengthening techniques. The fatigue behaviour of reinforced steel beams using nonprestressed and prestressed FRP composite is illustrated below.

### 7.1. Reinforced with Nonprestressed Polymer Composite

**Figure 6:**
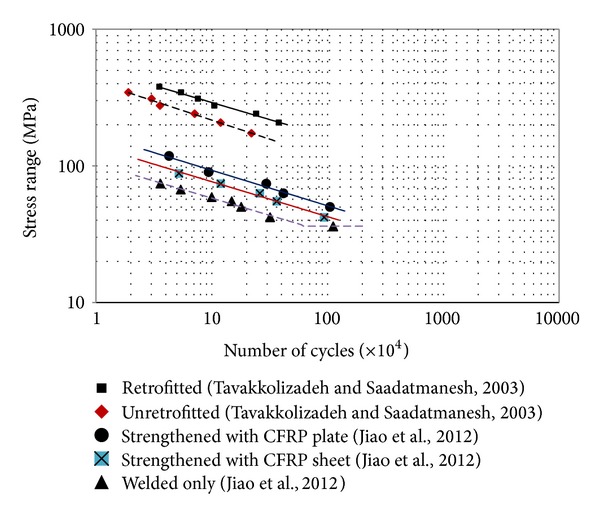
S-N curve for steel beam strengthened with nonprestressed CFRP [14, 40].

Hollaway and Head [122] indicated that unidirectional continuous fibre polymer composites, which essentially behave linearly up to failure level when loaded parallel to the longitudinal fibres, usually have good fatigue properties. Jiao et al. [40] compared the behaviour of notched steel beams using the welding method and retrofitted with CFRP plates and sheets, respectively, under flexural cyclic loads. In addition, two different epoxy adhesives, that is, Sikadur-330 and Araldite 420, were used in this test. The observations of the fatigue strength of the specimens reinforced with CFRP composites were extensively longer than that of specimens repaired with the welding method alone. It was observed that the strengthening system with one layer of CFRP plate adhesively bonded could extend the fatigue strength of steel beams about seven times compared to the beams only repaired with the welding method. In addition, the fatigue strength was extended about three times for beams strengthened with four layers of CFRP woven sheets, and no significant variation in fatigue strength could be observed for specimens strengthened using the epoxy adhesives of Araldite 420 and Sikadur-330. Mean S-N curves were obtained based on the test data (Figure 6), which can be used for predicting the fatigue strength of steel beams strengthened with similar CFRP composite materials.

Tavakkolizadeh and Saadatmanesh [14] demonstrated using the S-N curve that the application of CFRP strips could increase the fatigue lifetime of the structural elements more than three times (Figure 6). The design S-N curve for unreinforced and reinforced cut specimens was NS^3.54^ = 1.22 × 10^13^ and NS^3.96^ = 3.84 × 10^14^, respectively.

**Figure 7:**
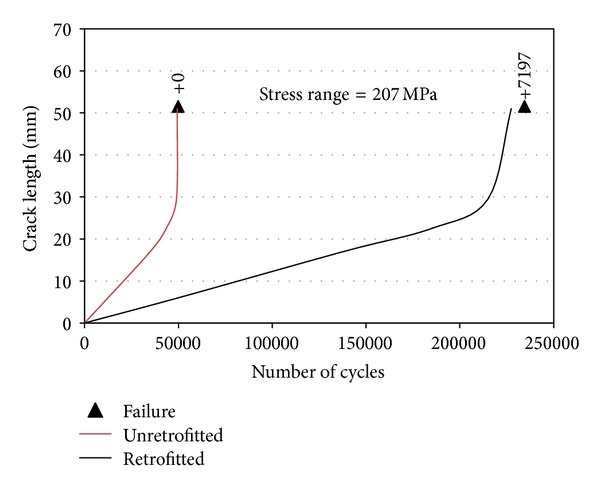
Fatigue crack growth curve for unreinforced and reinforced steel beams [14].

The slope of the S-N curves for the specimens (retrofitted and unretrofitted) in a log-log space was slightly smaller than the slope of the AASHTO design curves. They also observed that the CFRP patch not only decreased the crack growth rate but also was able to carry a few extra cycles even after the tension flange had completely cracked, especially under lower stress ranges (Figure 7).

Deng and Lee [55] reported the results of an experimental programme on small-scale steel beams reinforced by applying CFRP strips. From the tests results an S-N curve was obtained. The fatigue limit, that is, threshold of the S-N curve was about 30% of the ultimate static failure stress, which validates the fatigue limit recommended by the CIRIA Design Guidance [123]. To assess the fatigue bond resistance of a steel bridge girder reinforced with CFRP strips, Miller et al. [85] conducted two test programmes. First, they subjected seven small-scale, doubly reinforced specimens to cyclic loads at 82.7 MPa stress for 2.55 million cycles. All CFRP strips were found to remain fully bonded to the steel element without deterioration based on the strain data taken before and after the cyclic loading. Subsequently, two full-scale steel bridge girders retrofitted with CFRP were fatigued for ten million cycles at a constant stress range of 34 MPa. Throughout the tests, the CFRP strips were inspected and monitored for debonding, but none was detected. Therefore, the retrofitting technique was regarded as having good fatigue resistance.

Abed [124] investigated the effects of temperature on the adhesively bonded steel beams reinforced with CFRP composites. The adhesive materials showed a significant reduction in the fatigue life and failure load of the strengthened structures as the temperature reached the adhesive glass transition temperature (*T*_*g*_). Furthermore, Keller and Schollmayer [125] experimentally and numerically investigated the through-thickness performance of adhesively bonded FRP bridge decks and steel girders. They found that no stiffness degradation occurred for cyclic loading of up to 10 million cycles.

**Figure 8:**
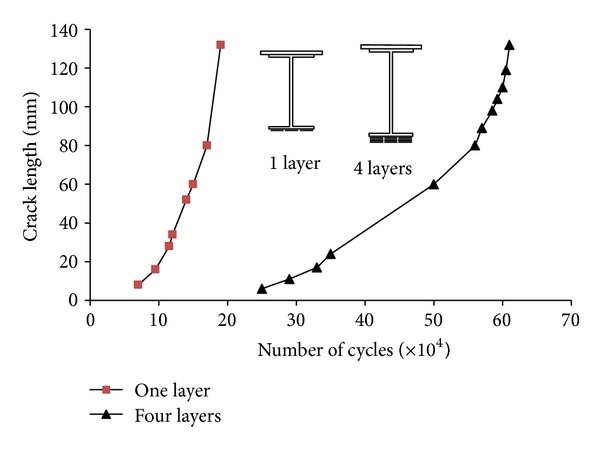
Effect of layers of strengthening material [38].

Wu et al. [38] investigated the fatigue behaviour of strengthened artificially notched steel beams including the effects of the configuration and the number of layers of HS-CFRP strips, the interface treatment of the SW-BFRP composites, and the type of materials. Compared to the traditional welded steel-plate approach, the experimental results showed that the use of a fibre-reinforced composite strip could not only delay crack initiation, decrease the crack growth rate, and prolong the fatigue life, but also reduce the residual deflection and stiffness decay. The rough surface of the SW-BFRP could extend the fatigue strength of steel beams more effectively than using SW-BFRP with a smooth surface. They also used high modulus CFRP strips as a reinforced material. HM-CFRP demonstrated the best strengthening performance; the fatigue strength of the steel beams could be improved significantly by increasing the number of layers of the strengthening material.  Figure 8 presents the crack expansion curves for four-layer and one-layer HS-CFRP. When the number of layers was increased from one to four, the crack initiation life and the fatigue lives were considerably enhanced (Figure 8). The plate configuration influences the fatigue strength.

### 7.2. Reinforced with Prestressed Polymer Composite

Although adhesively bonded FRP flexural strengthening techniques have been found to be an efficient approach to improve the lifetime of fatigued steel structures, there are relatively few studies that have applied prestressed CFRP strips to strengthen against cyclic loadings. Ghafoori et al. [64] developed a prestressed unbounded reinforcement (PUR) method and compared the effectiveness and feasibility of the approach with the prestressed bounded reinforcement (PBR) method. It could be used on heritage and historical structures where reversibility is important. The experimental test results for the strengthened beams using the PBR method showed a local strain concentration on the CFRP strip under the cracked section, while the PUR method had a uniform strain distribution along the CFRP strip. In addition, the fatigue performance of the unbonded reinforcement system was better at a high prestressing level of the CFRP without a substantial reduction in ductility.

**Figure 9:**
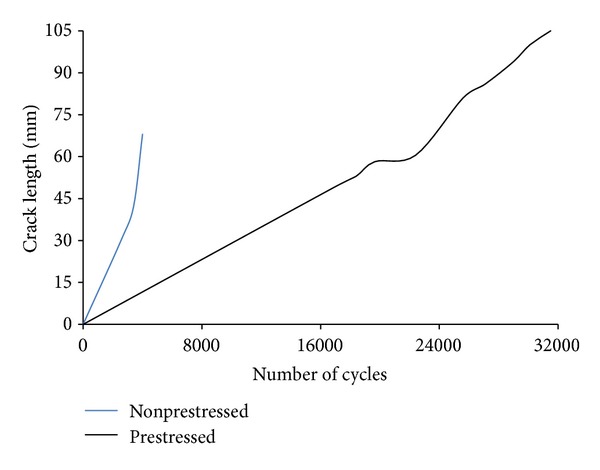
FCG curves for strengthened with nonprestressed and prestressed CFRP plates [63].

Ghafoori et al. [63] studied the behaviour of notched steel beams reinforced using prestressed and without prestressed bonded CFRP plates under cyclic loading. The experimental results showed that the fatigue strength of a beam reinforced using the prestressed CFRP plate increased more than five times that of an identical beam reinforced using nonprestressed CFRP plate (Figure 9). Both specimens were induced by a cyclic load range of 9–90 kN.

**Figure 10:**
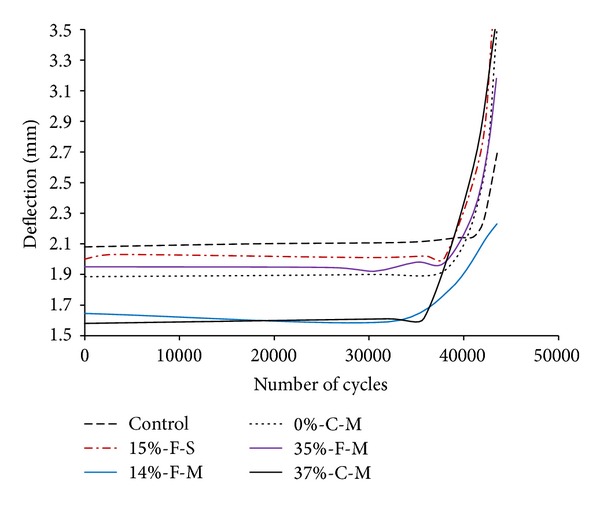
Deflection versus number of cycle curves for strengthened steel beam using 14%, 15%, 35%, 0%, and 37% prestressed CFRP plates [68].

Vatandoost [68] drew deflection range curves for W310 × 74 steel beam specimens strengthened with 14%, 15%, 35%, 0%, and 37% prestressed CFRP plates. The deflection range versus the number of cycle curves was drawn for the last 45000 cycles. Looking at Figure 10, it can be seen that (1) the highest deflection range belongs to the control beam while the lowest deflection range belongs to specimen 37%-C-M indicating the highest stiffness increase for that specimen. (2) The deflection ranges are dramatically increased at the end of the fatigue life. (3) The lower deflection range for the specimens with CFRP strips on the cover plates confirms the influence of the CFRP strip location on the specimen stiffness. In Figure 10, “F” indicates that the strips are bonded to the inner side of the flange, “C” indicates that the strips are attached to the cover plate, and “S and M” indicate the CFRP strip with a standard modulus and moderate modulus, respectively. The beams with reinforcement located on the cover plates showed a greater fatigue life improvement than those with reinforcement located on the inner side of the flange [68].

### 7.3. Fatigue in Hybrid-Joint and Nanoadhesive

In recent years, fatigue in hybrid adhesive joints, which combine a traditional mechanical joint and a layer of adhesive (bolted/bonded, welded/bonded, and rivet/adhesive), has attracted a considerable amount of researchers. This is due to their better fatigue performance compared to only mechanical joints or only bonded joints [126–130]. Furthermore, the use of nanoadhesives (carbon nanotubes, alumina nanoparticles, and quartz nanoparticles) is a new field of application to bonded joints and has the potential to improve their fatigue performance [127, 131].

## 8. Failure Modes of Reinforced Steel Beams

**Figure 11:**
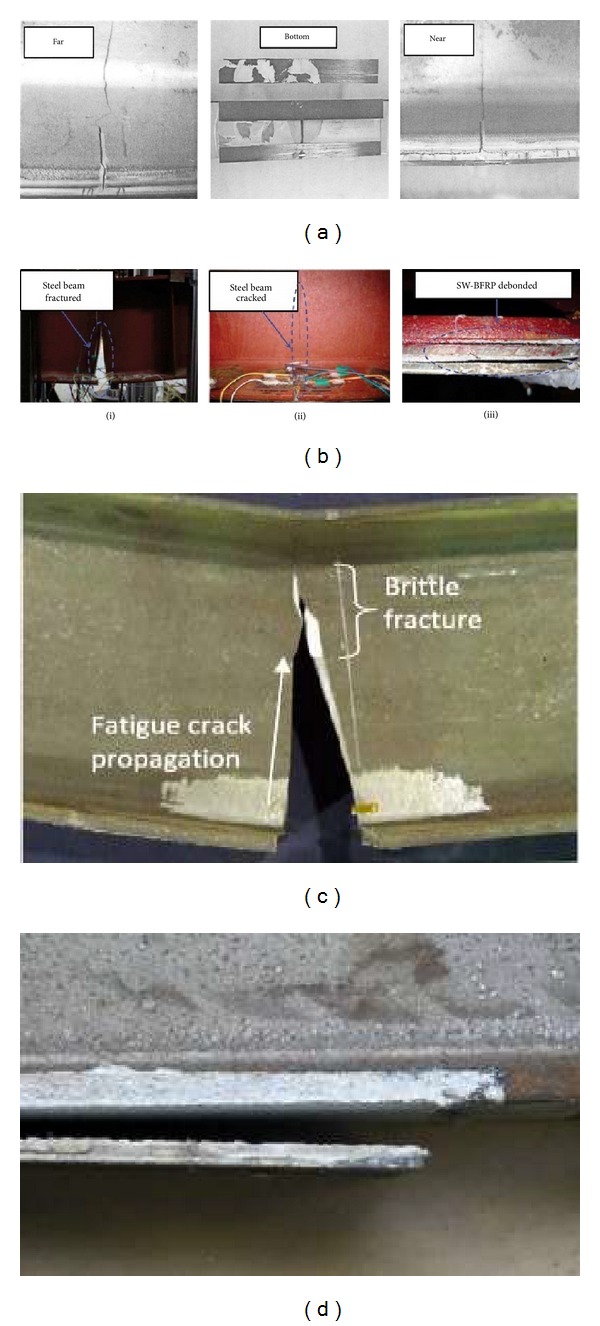
Failure modes of retrofitted beams using FRP plates (a) [14], (b) [38], (c) crack propagation and brittle web fracture [37], and (d) end debonding [55].

The deterioration of the steel bridge structural capacity over time may be due to corrosion, impact damage, and/or fatigue cracking [32, 51, 132]. The crack propagation and failure mode of FRP-strengthened steel structural techniques are normally different than concrete-FRP techniques [15]. Tavakkolizadeh and Saadatmanesh [14] studied the performance of steel I-beams with an edged notch in the tension flange and reinforced with a bonded CFRP patch under cyclic loading. They demonstrated that cracking started from the tip of one of the notches in the reinforced specimens and moved towards the web (Figure 11(a)). In Figure 11(a), the term “near” identifies the side that the crack started and “far” identifies the side that the crack terminated. After reaching the fillet section of the web, debonding started at the near edge of the CFRP patch. While the crack moved towards the far side, the debonding at the edge continued to grow. Even after the crack reached to the far cut end, the debonding remained fairly stable. Then the far edge of the CFRP patch started to debond. The reinforcement failed after around 50 mm of debonding on both sides.

Wu et al. [38] studied the fatigue behaviour of seven steel beams strengthened using four different strengthening materials. During the test, the fatigue crack always started from the notch tip at midspan as the loading cycles increased, mainly because of the stress concentration in this area. Initially, the cracks expanded slowly along the tension flange, and these cracks expanded at an increasing rate as the load cycled. For the steel beam retrofitted by a welded steel plate, the steel beam with plate fractured immediately when the crack moved through the tension flange, as shown in Figure 11(b)(i) and could not bear any further fatigue cycles. For specimens reinforced with FRP composite plates, extra loading cycles could still be sustained when the crack moved through the tension flange. As the load cycled, the crack expanded upwards on the web (Figure 11(b)(ii)) until debonding failure (Figure 11(b)(iii)). No fatigue rupture was sustained by the reinforced composite plate. Hence, the use of a reinforced composite plate can significantly improve the failure mode of the steel beam when compared to the use of a welded steel plate. The difference in failure modes was generally related to the fatigue strength and load-transferring mechanism of the reinforcement material. However, the failure modes depend on the elastic modulus of the FRP and the adhesive thickness [133].

Kim and Harries [37] investigated six beams intentionally damaged by notching the tension flange of the beam to appraise the static and fatigue performance of the beams with emphasis on local plasticity as well as the CFRP-steel interface. The retrofitted beam subjected to a high cyclic loading was observed to exhibit a large brittle fracture in the web (Figure 11(c)). The form of brittle fracture being addressed had been termed “constraint-induced fracture” and could occur without any noticeable fatigue crack growth and, more importantly, without any warning [134].

The FRP reinforcement ends and the regions where geometric discontinuities (cracks) take place are the most sensitive zones to fatigue damage of the adhesive joint, because of the stress concentration [28]. Deng and Lee [55] found similar crack initiation and propagation in the CFRP reinforced steel beams except one test specimen, which was investigated with a 92.6% load range that caused it to debond suddenly at one end after only 30 cycles, Figure 11(d). For the other specimens, the crack at each end grew quickly but then almost stopped after one of the two cracks had advanced past the midspan of the beams. For all the plates that had debonded, the cracking initiated from the middle of the spew fillet and then propagated to the steel surface and glue interface at a 45° angle.

**Figure 12:**
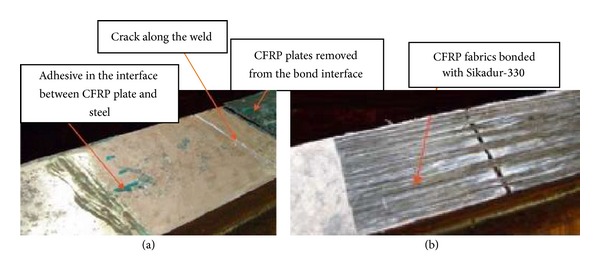
Debonding interface of retrofitted beams using (a) CFRP plates; (b) CFRP sheets [40].

Jiao et al. [40] observed various failure modes for beams strengthened using CFRP plates and sheets. A cut was made and welded along the bottom tension flange of each beam. For unstrengthened beams, a crack was initiated rapidly at the tip of the cut once the crack propagated along the weld on the beam soffit throughout the whole tension flange. It should be mentioned that the beam was considered to have failed when it lost the ability to carry the maximum load applied in every cycle due to severe crack propagation. For strengthened beams with CFRP plates, debonding occurred in the steel surface and epoxy bond interface (Figure 12(a)). Again, failure happened between the plies of the sheets in the strengthened beams as shown in Figure 12(b). The observation also confirmed that the performance of the CFRP plates to resist crack propagation was better than for the CFRP sheets under cyclic loading.

**Figure 13:**
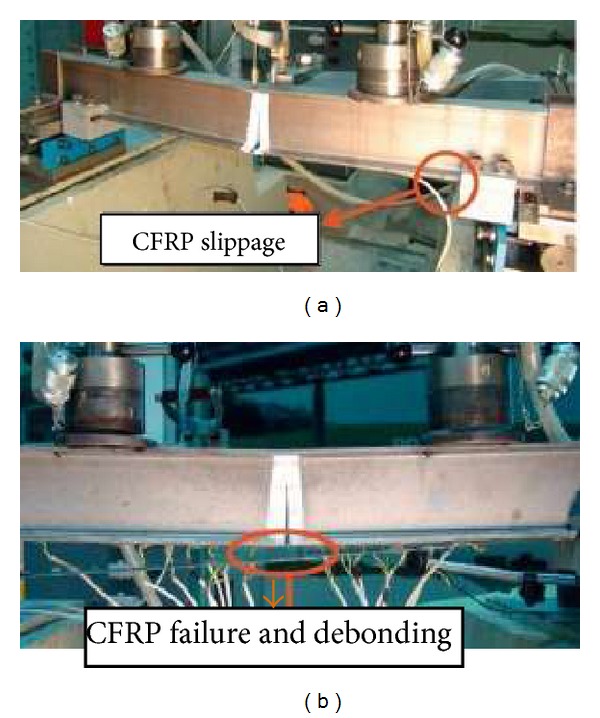
The failure modes for (a) PUR technique and (b) PBR technique.

The crack propagation rate depends on the stiffness of the FRP strip and, largely, on the prestressing force [73]. Ghafoori et al. [64] investigated the damaged steel beams reinforced with the PUR and PBR methods and obtained a similar load carrying capacity; however, the failure modes were different. In the PUR method the CFRP plate slipped in the mechanical anchorage system at the onset of the failure, while the CFRP in the PBR method arrived at its tensile strength in the cracked section and, finally, plate failure with debonding (Figure 13).

**Figure 14:**
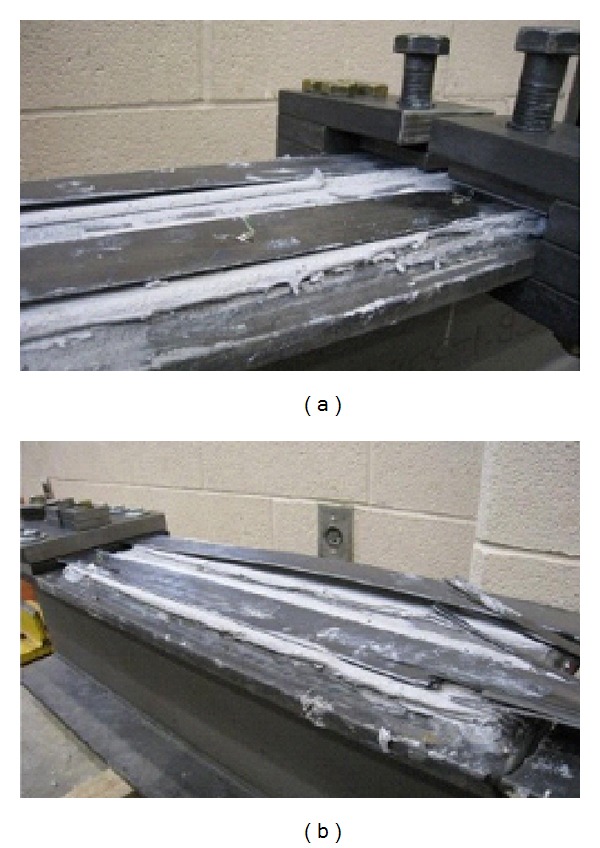
Performance of end anchorage after failure for steel beam strengthened with prestressed strips to the cover plate (a) removable end anchor and (b) fixed end anchor [68].

**Figure 15:**
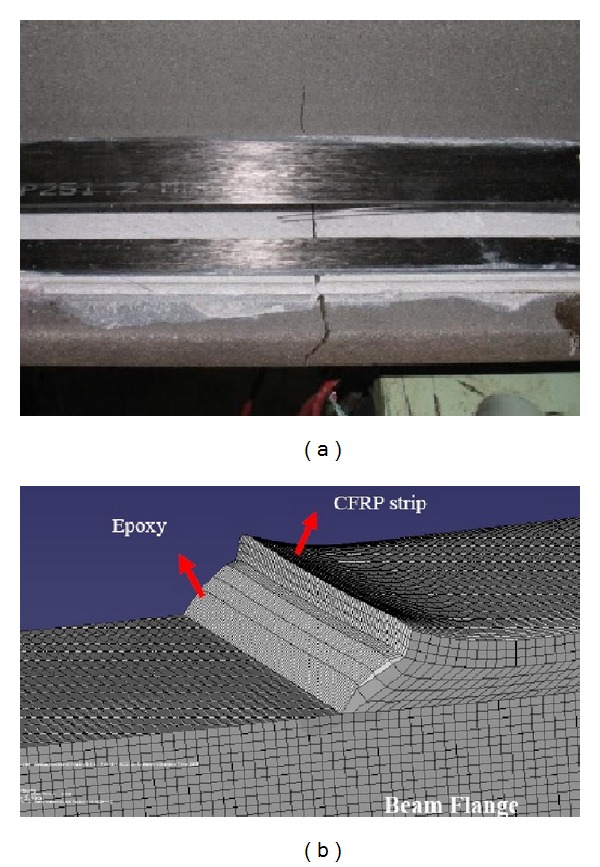
(a) Debonded strip rupture, (b) end debonding model using FEA [68].

The end anchoring technique mitigates the debonding of the FRP strips and maintains the prestressing force, hence also reducing the transfer length [68].  Figure 14 illustrates the effectiveness of the end anchoring technique in maintaining the CFRP prestress. The debonded CFRP strip rupture and the strip end debonding model using FEA is shown in Figure 15.

## 9. Conclusion

In this paper, detailed reviews on the relevant researches were investigated systematically and carried out in respect of CFRP/steel strengthening techniques under fatigue. Significant information and an explanation of the existing research on the fatigue behaviour of FRP-strengthened steel structures have been provided. The study also covered the surface treatment techniques, adhesive curing, and support condition under cyclic loading including fatigue performance, crack propagation, and failure modes with FE simulation of the steel bridge girders and structural elements.

The following conclusions can be drawn from the present study.

1. The remaining service life of bridge structures is limited by fatigue damage, and, in order to ensure the safety of the steel bridges, it is important to regularly check the structure to determine the existence of fatigue cracks.
2. The application of HM, HS, UHM, and prestressed CFRP and SW-BFRP strengthening composites not only delays the initial crack, reduces the crack growth rate, and extends the fatigue life, but also decreases the stiffness decay with residual deflection. In addition, strengthened with prestressed CFRP had the best strengthening effect.
3. The use of end anchorage prevented debonding of the CRRP strips at the beam ends by reducing the local interfacial shear and peel stresses.
4. Epoxy adhesive curing is needed for potential FRP strengthened structures. Cyclic loading during adhesive curing can decrease the fatigue life of the reinforced beam by reducing the bond strength.
5. Alteration in failure modes is mainly related to the fatigue strength and load-transferring mechanism of the strengthening material.
6. The prestressing force should be released very carefully to avoid debonding of the strengthened steel beams adhesively bonded with prestressed FRP.
7. Hybrid-joint, nanoadhesive, and carbon-flex can also be attractive for strengthening systems.

The following recommendations are suggested for future research in this area.

1. An effective surface treatment technique needs to be developed for use in practice that can prevent failure at the adhesive/steel interface under cyclic loading.
2. A comparative analysis of the fatigue behaviour of reinforced steel beams supported on a neoprene pad and steel plate in a simply supported manner should be investigated.
3. The effects of the thickness of the adhesive on the system require more investigation. In addition, the optimum adhesive thickness should be studied to slow down delamination.
4. The length of optimum reinforcement should be determined to retard fatigue failure like end debonding.
5. The influence of different environmental conditions must be studied to find the real fatigue behaviour of the strengthening scheme.
6. The effect of an acidic or alkaline environment on the fatigue strength has not been studied yet.
7. The fatigue behaviour of damaged steel structural elements reinforced with carbon-flex strengthening techniques should be investigated to enhance the cyclic load bearing capacity.
8. An appropriate rehabilitation method using FRP composites of welded steel structural elements under cyclic loading should be explored to retrofit welded steel bridge girders.
9. The application of nanoadhesives to FRP/steel bonded joints for rehabilitation of steel bridge girders to increase the fatigue life has not been investigated yet.
10. The use of hybrid-joints to strengthen steel structural elements with FRP requires further study.

## Figures and Tables

**Table 1:** Strengthening techniques.